# Patient and Health Care Professional Perspectives: A Case Study of the Lung Cancer Integrated Care Pathway

**DOI:** 10.5334/ijic.3972

**Published:** 2018-10-31

**Authors:** Francesca Bravi, Eugenio Di Ruscio, Antonio Frassoldati, Giorgio Narciso Cavallesco, Giorgia Valpiani, Anna Ferrozzi, Ulrich Wienand, Tiziano Carradori

**Affiliations:** 1 Research Innovation Quality and Accreditation Unit, S. Anna University Hospital of Ferrara, Ferrara, IT; 2 S. Anna University Hospital of Ferrara, Ferrara, IT; 3 Clinical Oncology, Department of Morphology, Surgery and Experimental Medicine, S Anna University Hospital, Ferrara, IT; 4 Department of Morphology, Experimental Medicine and Surgery, University of Ferrara, Ferrara, IT; 5 Clinical Audit, Healthcare Organisations Accreditation at Free Lance, Ferrara, IT

**Keywords:** Critical Pathways, Continuity of Care, Lung Cancer, Patient Centered Care, Health services research, CPSET questionnaire

## Abstract

**Introduction:**

The purpose of this study was to evaluate the perception of the quality of care, considering both patient experience and health care professionals’ perceptions as well as patient outcome measures of an integrated lung cancer pathway.

**Methods:**

A cross-sectional study was conducted in 2016 at Ferrara University Hospital, Italy. OPportunity for Treatment In ONcology (OPTION) questionnaires were administered to 77 patients, and the Care Process Self-Evaluation Tool (CPSET) questionnaires were given to 38 health care professionals. The effectiveness of the pathway was evaluated by analysing the tool’s positive impact on lung cancer surgery volume and 30-day mortality.

**Results:**

Seventy-seven patients were enrolled, and 38 health care professionals assessed the CPSET questionnaire. The highest scores were related to “respect” (100%), “satisfaction” (98.7%), and “trust” (97.4%) on the OPTION and to “patient-focused vision” (97.2%) and “patient engagement” (94.4%) on the CPSET. The lowest scores were related to “information” (26%) and “cooperation with general practitioner” (17.6%) on the OPTION and “cooperation between the hospital and primary care” (23.5%) for the CPSET. The outcomes analysis shows an increase in the volume of activity and a decrease in 30-day mortality after pathway implementation.

**Discussion:**

The lung cancer pathway is a patient-centred intervention that enables care to be shaped for patient needs in order to improve the quality and efficiency of service and clinical outcome.

## Introduction

Integrated care is a complex and comprehensive field that features many different approaches and, unfortunately, definitions [1]. In particular, the concept of integrated services has been defined by the World Health Organization as “the management and delivery of health services so that clients receive a continuum of preventive and curative services, according to their needs over time and across different levels of the health system” [2]. Common to this and other conceptual models is the recognition that integrated care is a “complex intervention” in which management and organizational processes to support integrated care occur simultaneously (or in due timing) at many levels [3]. The challenge for health systems is to navigate through the integrated care approach to respond to improved population health, patient experiences, and cost-efficiency [4].

One way to ensure coordination between health care professionals and facilities is the establishment of integrated care pathways (ICPs). The European Pathway Association (E-P-A) defines “care pathway” as “a complex intervention for the mutual decision making and organization of predictable care for a well-defined group of patients during a well-defined period. Defining characteristics of pathways include: an explicit statement of the goals and key elements of care based on evidence, best practice and patient expectations; the facilitations of the communication and coordination of roles, and sequencing the activities of the multidisciplinary care team, patients and their relatives; the documentation, monitoring and evaluation of variances and outcomes, and the identification of relevant resources” [5].

Among other objectives, ICPs are designed to provide “improved continuity of care” [6] and “improved clinician-patient communication and patient satisfaction” [7]. Defining characteristics of care pathways include “facilitation of communication, coordination of roles, and sequencing the activities of the multidisciplinary care team, patients and their relatives” [8]. In particular, the complexity of the challenges facing patients with cancer necessitates a comprehensive, multidisciplinary, and psychosocial approach to care [9].

Van Herck et al. reviewed the evidence on the real-world impact of ICPs, pointing out that their effects in terms of process flow and time schedules have not been adequately investigated; these were examined in only 13.5% of studies. Likewise, “goal setting, prioritizing and planning” were investigated in only 7.5% of cases, and service effects were almost always measured merely as “patient satisfaction” (18.5%) [10]. Another systematic review, by Allen et al., indicated that the effect of the interventions depended on a complex interrelation of directorial, coordinatory, organisational, decision-making, and accumulatory mechanisms. These authors also noted the added value of a well-designed ICP in terms of information transfer and patient-centred communication [11].

With regard to patients’ perception of such coordination, a review by Foglino et al. suggested that many studies report that specific organizational processes, such as information exchange among health care professionals, are associated with patient satisfaction, psychological and physical outcomes, and continuity of care. The patient experience is accepted as an important measure of performance for cancer care and is included in a large number of cancer care evaluations and report cards. When it has been assessed, the patient experience of integrated care appears to be related to important dimensions of performance, including patient satisfaction, quality of life, psychological and physical outcomes, empowerment, and continuity of care [12]. Nevertheless, to ensure that patients receive high-quality continuous care and a positive experience overall, it is therefore vital that processes for administration, communication, and coordination between services are understood and optimised [13]. In this regard, a recent qualitative analysis by Scotland’s National Cancer Patient Experience Survey suggested that patients with cancer would value greater integration of care from services involved in their treatment [14]. Indeed, efficacious care is distinguished not only by effective, patient-centred coordination of the care process, collaboration with primary care, and follow-up assessment but also effective communication, which should be perceived as such by patients and family [15]. That being said, the perception of the health care professionals themselves of the care processes they operate in has also been proposed to be an important dimension, enabling assessment of interprofessional teamwork, coordination, and communication [16,17].

The purpose of this study was to evaluate the perception of the quality of care provided in an integrated pathway. It is a complex intervention that could represent an effective model for the continuous, integrated management of the patient and improve patient outcomes. Our focus was therefore to investigate both patients’ and health care professionals’ perceptions of the organizational features of an ICP for lung cancer patients and to determine whether there is any correspondence between the two points of view. The effectiveness of the ICP was evaluated by analysing the tool’s positive impact on lung cancer surgery volume and 30-day mortality.

## Methods

This is a cross-sectional study in patients and health care professionals involved in the Lung Cancer ICP adopted by Ferrara University Hospital and approved by the University Hospital directorate in February 2012. This ICP, initiated in 2011, was set as part of a new strategy for overseeing clinical processes and improving quality of care. A workgroup involving various health care professionals (surgeon, pneumology specialist, radiotherapy oncology specialist, oncologist, radiologist, nuclear medicine specialist, anaesthesiologist, anatomopathologist, and a new professional figure, the case-manager nurse) was set up to oversee the transition, and this team meets weekly to discuss each case and define the responsibilities of each involved member.

**Figure 1:**
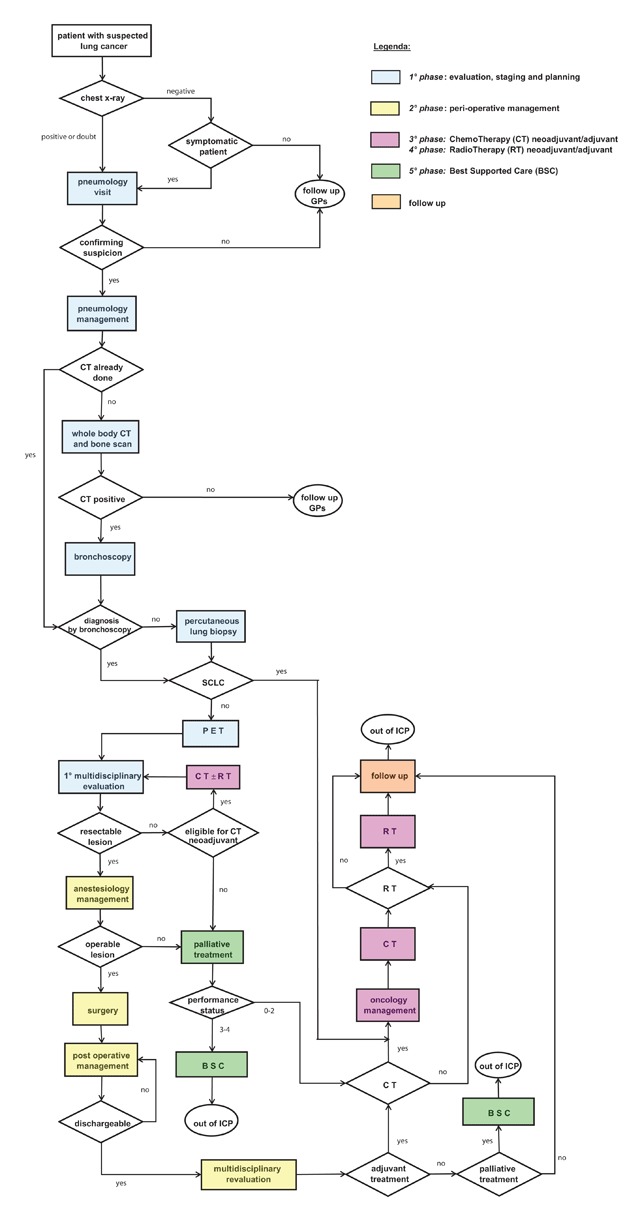
Flow chart ICP.

Multidisciplinary discussion provides an evidence-based approach to treat patients, and care is standardized according to international guidelines. A positive environment allows clinicians to share their experience and knowledge [18]. The ICP was set up by a methodology adhering to the cardinal principles of the E-P-A with regard to patient-centred care [19], and indicators designed to measure effectiveness and timeliness, as well as other objective aspects of the care pathway as a whole, are regularly monitored (Figure 1).

From August to November 2016, questionnaires were filled out by ICP staff and patients from the Ferrara S. Anna University Hospital catchment area (which caters to roughly 350,000 inhabitants), located in northeast Italy. Specifically, patients were administered the Opportunities for treatment in Oncology (OPTION) self-report questionnaire [21], while the staff involved in the various stages of the ICP filled in the self-report Care Process Self-Evaluation Tool (CPSET) [15,17,21].

### Outcome measures

We used the National Outcome Programme (http://pne2017.agenas.it/) of the Italian National Health Service to measure lung cancer surgery volume and 30-day mortality in the Ferrara University Hospital catchment area from 2009 to 2016 [22].

### Data collection

#### OPTION Questionnaire

The OPTION questionnaire, validated in Italy [20,23], analyses the continuity of care [24,25] perceived by patients with cancer in terms of three major domains: information, management, and relational. There are 33 multiple-choice items on the questionnaire and an open-answer section at the end, which gives patients an opportunity to freely express any suggestions or proposals for improvement that they may have. The first 19 multiple-choice items explore the patients’ perceptions of the continuity of their care within the ICP. They are measured as anchors on a 5-point Likert scale, in which 1 = *never*, 2 = *almost never*, 3 = *sometimes*, 4 = *often*, and 5 = *always*. One of these items specifically investigates the presence or absence of a “care coordinator” (a specialist, general practitioner [GP], or nurse, designated as a point of reference) within the ICP; if they answered yes to this question, they were asked to provide responses to four more items (Likert scale from 1–5) exploring the degree to which this person facilitated their access to services, maintained contact with other caregiving professionals, and knew their individual medical history. The second part of the OPTION questionnaire was designed to collect data on patients’ gender, age, time elapsed since diagnosis, and the accessibility of the ICP (screening, private testing, referred by a GP, etc.). It also contained an item investigating whether a patient had sought a second opinion. The questionnaire was administered to adult patients diagnosed with lung cancer and enrolled in the ICP. Patients with evident cognitive disorders were excluded. Questionnaires were administered anonymously at the University Hospital Oncohaematology Clinic during the chemotherapy stage or follow-up. The case-manager nurse drew up the list of patients to survey and, together with the care manager, sought their informed consent for participation in the study during scheduled day-hospital visits. Participants were administered the questionnaire by purpose-trained hospital staff from other departments, who read the items to participants in the event of any reading difficulties. Otherwise, patients completed the questionnaire without any intervention from the staff member, in roughly 15 minutes.

#### CPSET Questionnaire

The CPSET questionnaire [15,21] investigates the perception of different health care professionals involved in the various stages of the ICP. It serves to identify the expected impact of the ICP on its process outcomes. It has been shown to be an effective tool for improving the performance of a multiprofessional, multidisciplinary team and is available in Dutch (original), English, French, Spanish, and Norwegian [21,26,27]. The content, face, construct, and criterion validity and the reliability of this tool are excellent and have been described elsewhere [21]. The CPSET questionnaire asks health care professionals to rate 29 items on a 10-point ordered Likert scale, ranging from *totally disagree* (1) to *totally agree* (10).

The items explore 5 domains, specifically patient-focused organisation (PO; six items), coordination of the care process (COR; seven items), communication with patient and family (COM; four items), collaboration with primary care (SE; three items), and monitoring and follow-up of care process (OP; nine items).

The questionnaire was filled in by a total of 38 named health care professionals working within the ICP; it took roughly 15 minutes to complete.

#### Statistical Analysis

Responses of patients and health care professionals were recorded in two distinct Excel files. The patients’ anonymity was protected by assigning each compiled questionnaire a progressive personal ID number. Although health care professionals’ identities were not hidden from the investigators during data collection, their responses were anonymised when recorded on the spreadsheet. The ages of the patients and health care professionals’ were expressed as mean ± standard deviations and range (minimum and maximum age values), while categorical data (sex, education, cancer type, time from diagnosis, care coordinator, ICP entry mode, and request for a second opinion) were expressed as absolute values and percentages. The chi-square test was used to compare percentages, and the Student’s *t* test was used to compare the mean ages of the patient and healthcare professional groups. The OPTION and CPSET questionnaires relied on two different ranges of measurement (Likert scale from 1–5 and Likert scale from 1–10, respectively) and no shared factors or constructs. Hence, to achieve the objective of the study (i.e., comparison of the two sets of responses), these results, albeit quantitative, needed to be interpreted from a purely conceptual, qualitative standpoint. As questionnaire items are scored on an ordinal scale, on 5- and 10-point Likert scales, data are presented as percentage values (data no show). For analysis, we combined scores of 1 and 2 on the OPTION questionnaire and 1 to 4 on the CPSET questionnaire to represent the “worst scores”; similarly, we combined the scores of 4 and 5 and from 7 to 10 (very good to excellent) to represent the “best scores.” These data are presented as percentage values. To provide a visual comparison of patients’ and health care professionals’ perceptions of the organisational features of the lung cancer ICP, we plotted both sets of worst and best scores on the same graph. Statistical analysis was performed using SPSS 23.0 (IBM, New York, NY, USA), and the significance threshold was set at *p* = 0.05.

## Results

A total of 77 OPTION and 38 CPSET questionnaires were administered to those being treated and those working in the lung cancer ICP, respectively. No patient refused to complete the survey, but the response rate for the first 19 items that explore the patients’ perception of the continuity was 69%. Four health care professionals refused to complete the CPSET questionnaire because they had just joined the lung cancer ICP. Moreover, two professionals returned the blank CPSET questionnaire, without providing responses. Thus, the response rate to the CPSET was 80.6%. About sixty percent (60.5%) of the patients included in the study were female and ranged widely in age (mean, 45 years). Men were predominant in the health care professional group (63.2%), which had an average age of 52 years. Statistically significant differences between the two study groups were found not only for sex and age but also for the level of education (*p* < 0.001; Table 1).

**Table 1:** Demographic characteristics from OPTION and CPSET questionnaire.

Characteristic	Patients *n* = 77	Health care Professionals *n* = 38	*p*-value

**Gender, n (%)**			0.017
Male	30 (39.5)	24 (63.2)	
Female	46 (60.5)	14 (36.8)	
*Valid cases n (%)*	*76 (98.7)*	*38 (100.0)*	
**Age, mean ± SD (min–max)**			
Male	67 ± 8 (50–79)	52 ± 10 (29–65)	<0.001
Female	67 ± 10 (38–83)	47 ± 10 (27–63)	<0.001
*Valid cases n (%)*	*76 (98.7)*	*36 (94.7)*	

All health care professionals had a degree; 26.7% of patients were elementary school graduates, 46.7% were middle school graduates, 25.3% were high school graduates, and 1.3% had a degree. With regard to civil status, 69.3% of patients were married or cohabiting, 14.7% were single, 12% were widowed, and/or 4% were divorced (Table 2). A total of 41.3% of cases were referred to the ICP by a GP, while 28% of the cases were referred by private practitioners. Forty patients, or 52.6% of respondents, said that less than a year had elapsed since their diagnosis of pulmonary neoplasia, while 6.6% (five patients) had been diagnosed more than 5 years previously. The oncologist was considered by patients as the care manager among those involved in their care (surgeon, radiotherapy oncology specialist, GP, nurse). In fact, of the 41 patients who identified such a figure, 87.8% stated that their oncologist was their care manager, whereas one patient stated that the nurse case manager was their major contact. Seventy-two percent of respondents stated that they did not seek a second opinion from another health care professional, while 15% asked for a second opinion from a private specialist and 8% requested a second opinion free of charge.

**Table 2:** Patient characteristics from OPTION questionnaire.

Characteristic	Patients *n* = 77

**Marital status, n (%)**	
Single	11 (14.7)
Married or cohabiting	52 (69.3)
Divorced	3 (4.0)
Widowed	9 (12.0)
*Valid cases*	*75 (97.4)*
**Education, n (%)**	
No qualification	0 (0)
Elementary school	20 (26.7)
Middle school	35 (46.7)
High school	19 (25.3)
Degree	1 (1.3)
Postgraduate	0 (0)
*Valid cases*	*75 (97.4)*
**Time from diagnosis, n (%)**	
Less than 1 year	40 (52.6)
1–2 years	22 (28.9)
3–4 years	9 (11.8)
More than 5 years	5 (6.7)
*Valid cases*	*76 (98.7)*
**Care coordinator, n (%)**	
Yes	41 (55.4)
No	33 (44.6)
*Valid cases*	*74 (96.1)*
**Mode of ICP entry, n (%)**	
Screening	6 (8.0)
Illness	31 (41.3)
Private testing	22 (29.3)
Other	16 (21.4)
*Valid cases*	*75 (97.4)*

To rank the “worst scores” (1–2 points on Likert scale for OPTION and 1–4 points for CPSET) and “best scores” (4–5 points on Likert scale for OPTION and 7–10 points for CPSET) on the two questionnaires, percentage values were calculated. Each point represents a pair of percentage values that create a coordinate in the graph that derives from the proportion (percentage) of responses from the two questionnaires, best scores (4–5 points for OPTION and 7–10 points for CPSET) or worst scores (1–2 points for OPTION and 1–4 points for CPSET). In Table 3, we present in descending order the frequency distribution for OPTION and CPSET items.

**Table 3:** Frequency distribution items OPTION and CPSET.

WORST SCORES *(in descending order)*				BEST SCORES *(in descending order)*			

*OPTION questionnaire* 1–2 points on Likert scale	*%*	*CPSET questionnaire* 1–4 points on Likert scale	*%*	*OPTION questionnaire* 4–5 points on Likert scale	*%*	*CPSET questionnaire* 7–10 points on Likert scale	*%*

ITEM 9 – Information on social and personal changes	26.0	SE2 – Good cooperation exists between the hospital and primary care	23.5	ITEM 15 – Treated with respect by staff	100	PO1 – A patient focused vision exists within the organisation	97.2
ITEM 4 – Cooperation between hospital staff and GP	17.6	SE3 – In complex care situations consultation takes place between the physician/surgeon and general practitioner	23.5	ITEM 18 – Satisfied with care received	98.7	COM4 – The patient is explicitly asked for his consent with regard to the proposed care	94.4
ITEM 10 – Information on symptoms and lifestyle	16.0	PO6 – There is a clear vision of policy regarding care throughout the entire hospital	14.3	ITEM 13 – Trust in staff	97.4	PO3 – The care process coordinator has a patient focused vision	94.3
ITEM 11 – Requests for information missing from medical records	12.0	SE1 – Primary care is considered by the hospital to be an equal partner	11.4	ITEM 1 – Easy to get an appointment	96.1	PO4 – Patient communication is considered to be important within the organisation	94.1
ITEM 12 – Perceived familiarity of staff	8.1	PO5 – The organisational structure is patient focussed	11.1	ITEM 6 – Information on tests and examination	95.9	COR4 – Concrete agreements are made within the care process	91.4
ITEM 8 – Information on treatment side effects and physical changes	8.0	COM1 – Within the care process time is explicitly provided to listen to the patient and his family	11.1	ITEM 5 – Explanation of care pathway steps	94.7	PO2 – Quality of care is the priority within the organisation	88.9
ITEM 16 – Lack of identification of a personal care coordinator	6.7	OP2 – Whether the care provided is tailored to the patient’s needs is systematically monitored/followed-up	8.6	ITEM 3 – Cooperation among professionals	94.6	COR1 – Agreements are observed	88.9
ITEM 17 – Sense of physical and emotional abandonment	6.7	COM2 – Time is explicitly scheduled within the care process for communications between healthcare professional and patient	8.3	ITEM 14 – Listening and emotional support received	92.1	COR2 – All team members are familiar with the various steps in the care process	88.9
ITEM 19 – Involved in care-related decision-making	5.2	COR3 – There is an optimum timing of activities within the care process	5.6	ITEM 17 – Sense of physical and emotional abandonment	88.0	COR3 – There is an optimum timing of activities within the care process	88.9
ITEM 7 – Information on treatments	1.4	COR5 – Team members consider themselves to be engaged in the organisation of the care process	5.6	ITEM 8 – Information on treatment side effects and physical changes	86.7	COR6 – Patients/family are provided with candid (frank; open; straightforward) information regarding their health	88.9
ITEM 3 – Cooperation among professionals	1.4	COM3 – Within the care process there is provision for sufficient time to provide information	5.6	ITEM 12 – Perceived familiarity of staff	86.5	COR7 – Discharge is communicated in a timely manner to the patient and family so that they can take necessary measures	88.9
ITEM 6 – Information on tests and examination	1.4	OP9 – The progress in the care process is continuously monitored/followed-up and adjusted	5.6	ITEM 7 – Information on treatments	86.3	OP1 – When (re)designing the care process quality indicators are formulated	88.9
ITEM 5 – Explanation of care pathway steps	1.3	PO4 – Patient communication is considered to be important within the organisation	2.9	ITEM 16 – Lack of identification of a personal care coordinator	85.3	OP4 – The goals of the care process are described explicitly	88.9
ITEM 1 – Easy to get an appointment	1.3	OP3 – Within the care process patient satisfaction is monitored/followed-up systematically	2.8	ITEM 19 – Involved in care-related decision-making	84.4	COR5 – Team members consider themselves to be engaged in the organisation of the care process	83.3
		OP5 – Within the care process monitoring/follow-up is performed to verify whether planned activities are actually performed	2.8	ITEM 11 – Requests for information missing from medical records	78.7	COM3 – Within the care process there is provision for sufficient time to provide information	83.3
		OP7 – Variances can be monitored within the care process	2.8	ITEM 10 – Information on symptoms and lifestyle	72.0	OP2 – Whether the care provided is tailored to the patient’s needs is systematically monitored/followed-up	82.9
		OP8 – Within the care process risks of complications are monitored/followed-up systematically	2.8	ITEM 4 – Cooperation between hospital staff and GP	71.6	PO5 – The organisational structure is patient focussed	80.6
				ITEM 9 – Information on social and personal changes	64.4	OP6 – Outcomes are systematically monitored/followed-up	80.6
						OP9 – The progress in the care process is continuously monitored/followed-up and adjusted	80.6
						OP5 – Within the care process monitoring/follow-up is performed to verify whether planned activities are actually performed	75.0
						OP8 – Within the care process risks of complications are monitored/followed-up systematically	75.0
						COM2 – Time is explicitly scheduled within the care process for communications between healthcare professional and patient	72.2
						OP7 – Variances can be monitored within the care process	72.2
						OP3 – Within the care process patient satisfaction is monitored/followed-up systematically	63.9
						PO6 – There is a clear vision of policy regarding care throughout the entire hospital	60.0
						COM1 – Within the care process time is explicitly provided to listen to the patient and his family	58.3
						SE1 – Primary care is considered by the hospital to be an equal partner	57.1
						SE2 – Good cooperation exists between the hospital and primary care	44.1
						SE3 – In complex care situations consultation takes place between the physician/surgeon and general practitioner	35.3

Four items (ITEM 13 – Trust in staff, ITEM 14 – Listening and emotional support received, ITEM 15 – Treated with respect by staff, ITEM 18 – Satisfied with care received) from OPTION questionnaire and twelve items (PO1 – A patient focused vision exists within the organisation, PO2 – Quality of care is the priority within the organisation, PO3 – The care process coordinator has a patient focused vision, COR1 – Agreements are observed, COR2 – All team members are familiar with the various steps in the care process, COR4 – Concrete agreements are made within the care process, COR6 – Patients/family are provided with candid (frank; open; straightforward) information regarding their health, COR7 – Discharge is communicated in a timely manner to the patient and family so that they can take necessary measures, COM4 – The patient is explicitly asked for his consent with regard to the proposed care, OP1 – When (re)designing the care process quality indicators are formulated, OP4 – The goals of the care process are described explicitly, OP6 – Outcomes are systematically monitored/followed-up) from CPSET questionnaire are not listed in Table 3 because they have zero response rates.

**Figure 2:**
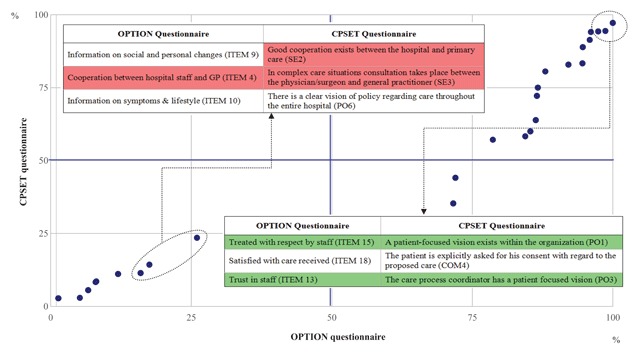
High and low scores for OPTION and CPSET questionnaire.

Both sets of responses revealed patient-centred care as one of the strengths of the ICP; patients generally reported feeling respected and placing their trust in the health care professionals. Specifically, high scores on the OPTION questionnaire were most often given for item 15, treated with respect by staff (100%); item 18, satisfied with care received (98.7%); and item 13, trust in staff (97.4%). On the CPSET questionnaires, staff tended to give the highest scores for items: PO1, a patient-focused vision exists within the organization (97.2%); COM4, the patient is explicitly asked for his consent with regard to the proposed care (94.4%); and PO3, the care process coordinator has a patient-focused vision (94.3%). The lowest OPTION questionnaire percentages (1–2 points on Likert scale) were most often given for: item 9, information on social and personal changes (26.0%); item 4, cooperation between hospital staff and GP (17.6%); and item 10, information on symptoms and lifestyle (16%). Similarly, lower CPSET scores (1–4 points) were most frequently given for items SE2, good cooperation exists between the hospital and primary care (23.5%); SE3, in complex care situations, consultation takes place between the clinical/surgeon and GP (23.5%); and PO6, there is a clear vision of policy regarding care throughout the entire hospital (14.3%) (Figure 2).

### Outcome measures

**Figure 3:**
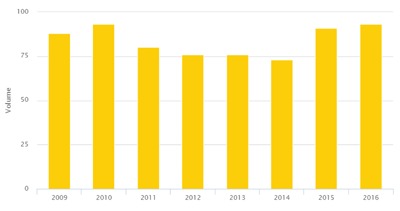
Lung cancer surgery volume.

**Figure 4:**
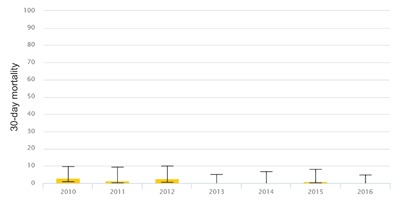
Lung cancer: 30-day mortality.

The outcome analysis shows an increase in the volume of activity and a decrease in 30-day mortality after ICP implementation (Figures 3 and 4).

## Discussion

The results of the study reveal that both patients and health care professionals consider it important to focus on the individual, and patients in particular feel that it is important for them to be treated with respect and to have confidence in the staff who care for them. Likewise, health care professionals believe that a patient-focused vision is essential for the organisation (Figure 2). These results are reflected in the work of Busetto et al., in which patients’ and health care professionals’ experience of ICP is a milestone for a person-centred approach [4]. Analysis of the two sets of responses shows that there are points of overlap regarding the perceived strengths and weaknesses of this ICP. In particular, both sets of respondents validated the efforts made to respect (OPTION item 15) and build trust with the patient (OPTION item 13), rather than viewing the patients as mere recipients of care. In other words, both patients and health care professionals perceived that the organization of the ICP was patient centred (CPSET item PO1). Similarly, both patients and health care professionals perceived similar weaknesses, in particular a relative lack of cooperation between hospital staff and GPs (OPTION item 4, CPSET items SE2 and SE3); these responses suggest that the GP is relegated to a marginal role within the ICP, and cooperation between the two care providers should be improved (Figure 2).

The care is patient-centred from both the patient’s and physician’s point of view, but the patients note that little or unuseful information was given about the changes that illness or care will bring and that there is not enough collaboration/integration between hospital staff and the GP. The hospital staff recognize only the lack of integration with GPs. Recent literature suggests that to improve continuity of care and integration between the hospital and primary care, navigator programs are needed in which the case manager is the figure identified [28,29]. Indeed, our results show that the figure identified as the care manager is the clinician responsible for the case. A public hospital “as a whole, in this view, is constructed to produce specific services that have ‘use value’ to their recipients.” In particular, the “use value” is a reconfiguration in political-economic terms of the concept of “public needs.” In this clinical setting patients being happy when a doctor as to see them [30].

There were also some differences between the two sets of responses; of particular interest is the different importance given to communication by patients and health care professionals. The term *communication* comprises various facets—not only verbal transmission of the diagnosis, test results, and discharge dates but also informing patients of potential side effects of treatment, recommended lifestyle changes, and the effects of the diagnosis on their personal and social lives. These aspects are investigated specifically in OPTION item 8 (information on treatment side effects and physical changes [8.0%]) and OPTION item 10 (information on symptoms and lifestyle [16.0%]), which placed very low in patients’ rankings. This indicates that the importance placed on these communicative aspects is not being adequately matched by the information given by health care professionals. Indeed, the health care professionals placed patient communication items high on the CPSET ranking. It is therefore clear that the message is not being fully conveyed, and more attention needs to be paid to improve this vital aspect of care provision and that social and psychological factors of illness should be discussed in more depth with patients.

The authors of the study that validated the OPTION questionnaire came up with a five-factor scale to evaluate the responses, specifically factor 1, trustful relationship with health care staff; factor 2, information on care pathway; factor 3, information on physical changes related to the illness; factor 4, feelings of abandonment; and factor 5, collaboration among health care professionals [20]. In our study, trust in the health care staff was important for almost all patients (97.4%); as previously described in the literature [31], our results also show that the involvement of patients in care processes affects their satisfaction with the treatment received (98.7%). Health care professionals also believed in the importance of focusing on patient-centred care (97.2%), stating that among the skills required of health care professionals is the ability to empathise, listen, and treat patients with respect (100.0%).

Inevitably, the results of this study need to be interpreted in light of several limitations, which may have affected its methodological reliability. First, we do not have pretest information on patients’ and health care professionals’ perceptions of the care process before ICP implementation. Second, GPs, although essential to continuity of hospital and primary care integration, were not surveyed. Third, as the organisation of an ICP is likely to vary from facility to facility, results are not generalizable to other settings, even though a recognised methodology (E-P-A) was used to set up the ICPs. Moreover, as already mentioned above, the lack of parallels in Likert scoring and questionnaire items makes quantitative comparative analysis of the two sets of responses impossible. Aside from these methodological limitations, it should be noted that our comparison of the two perspectives, and the weaknesses and strengths highlighted by patients and health care professionals, is not intended to be interpreted in absolute terms; indeed, patients and health care professionals have very different roles and therefore very different experiences within the ICP. That being said, our investigation has enabled us to assess several important aspects of the ICP, namely, coordination, a patient-focused organisation, and communication, from both perspectives. Both sets of participants were recruited within the same time frame and setting; in other words, the patient participants were being treated by the health care professional interviewees at the time of the survey, and therefore, both were able to provide a different point of view of the same experience.

This approach enabled us to acquire a general overview of the functioning of the ICP—judged largely positive by both types of users—and provided a springboard for potentiating those aspects of care that require some improvement, namely, those aspects of communication that may be judged as “secondary” by the health care professionals but are deemed vital by the patients whom they treat.

ICPs aim at sharing decision-making processes and the organisation of care for a specific group of patients in a well-defined period of time. Our study results show that patients perceived an increase in the quality of care, an increase in health care professionals’ positive perceptions of the organizational features of an ICP, and an improvement in patient outcomes (lung cancer surgery volumes and 30-day mortality).

## Conclusions

This study is intended to inform policy makers and health care management personnel who are charged with the ongoing evaluation of complex interventions and the organisation of good quality care processes. Care pathways are complex interventions that include teamwork, the practical organisation of care, and the integration of different care settings. One of the objectives of such pathways is to improve the quality and efficiency of the care provided; this is deeply linked to the involvement of patients and health care professionals, which can be ensured through the use of such validated tools to shed light on their respective needs and perceptions.

ICPs are widely recognised in the medical literature as one of the main tools to make clinical networks operational, that is, to design and structure care processes by focusing on patients’ needs, thus facilitating the quality of care promotion [5]. With respect to the ICP implementation within a clinical network, the effectiveness of an ICP as a support tool for multidisciplinary teamwork and its positive impact on patient outcomes should not be underestimated [32,33].

We show that in an ICP, the views of patients and health care professionals overlap on aspects considered important, namely, a person-centred approach. Their perception of weaknesses is also similar, in particular a relative lack of patient communication and cooperation between hospital staff and GPs.

Lung cancer ICP is a patient-centred intervention that allows shaping care to patient needs, improving quality and efficiency of service and clinical outcome.
